# Supplementary material for: Exploring the Role of Community Exercise Rehabilitation Centers through the Rehabilitation Experiences of Patients with Musculoskeletal Disorders: A Qualitative Study
Source: Healthcare (Basel). 2023 Dec 30;12(1):92. doi: 10.3390/healthcare12010092 (PMC10778574; doi:10.3390/healthcare12010092)
Supplement: Supplementary file 1 [file healthcare-12-00092-s001.zip › healthcare-2762115-supplementary.pdf]

healthcare MDPI

A Semi-Structured Interview Questionnaire

Article title: Exploring the Role of Community Exercise Rehabilitation Centers through the Rehabilitation Experiences of Patients with Musculoskeletal Disorders: A Qualitative Study

- Do you have prior experience in undertaking exercise rehabilitation for functional recovery (pain relief) before participating in Living Lab?
- What efforts have you made in the past for health promotion?
- What was your perception of local community exercise rehabilitation centers before joining the Living Lab?
- Through what means did you decide to participate in the Living Lab, and what motivated your decision to join?
- What was your initial impression when you first visited the Living Lab?
- How did the exercise instructor guide the exercises?
- Do you feel that the exercise has been beneficial for knee pain or overall health?
- Are you motivated to initiate physical activity and make a conscious effort towards health maintenance?
- If you were to highlight the most significant effect, what would it be?
- Do you have intentions to continue participating in exercise after the conclusion of the exercise rehabilitation services?
